# Significant improvement of miRNA target prediction accuracy in large datasets using meta-strategy based on comprehensive voting and artificial neural networks

**DOI:** 10.1186/s12864-019-5528-1

**Published:** 2019-02-27

**Authors:** Bi Zhao, Bin Xue

**Affiliations:** Department of Cell Biology, Microbiology and Molecular Biology, School of Natural Sciences and Mathematics, College of Arts and Sciences, University of South Florida, Tampa, FL 33620 USA

**Keywords:** miRNA target, Meta-strategy, Dual-threshold, Two-step significance voting, Artificial neural network

## Abstract

**Background:**

Identifying mRNA targets of miRNAs is critical for studying gene expression regulation at the whole-genome level. Multiple computational tools have been developed to predict miRNA:mRNA interactions. Nonetheless, many of these tools are developed in various small datasets, which each represent a limited sample space. Thus, the prediction accuracy of these tools has not been systematically validated at a larger scale. Accordingly, comparing the prediction accuracy of these tools and determining their applicability become challenging. In addition, the accuracy of these tools, especially in large datasets, needs to be improved for broader applications.

**Results:**

In this project, a large dataset containing more than 46,600 miRNA:mRNA interactions was assembled and split into eleven subsets based on the availability of prediction scores of four individual predictors, which are miRanda, miRDB, PITA, and TargetScan. In each of these subsets, the predictive results of four individual predictors were integrated using decision-tree based artificial neural networks to make the meta-prediction. The decision-tree is used here to sort the predictive results of four individual predictors, and artificial neural networks are applied to make meta-prediction based on the outputs of individual predictors. In the decision tree, dual-threshold and two-step significance-voting were incorporated, information gain was analysed to select threshold values. The prediction performance of this new strategy was improved significantly in most of the eleven datasets comparing to the individual predictors and other meta-predictors, such as ComiR, under multi-fold cross-validation, as well as in independent datasets. The overall improvement of prediction accuracy in independent datasets is at least 9 percentile points comparing to the other predictors, and the percentage of improvement of F1 and MCC scores is at least 40% compared to the other predictors.

**Conclusions:**

The combination of dual-threshold, two-step significance-voting, and analysis of information gain is very effective in optimizing the outcome of decision-tree, and further integration with artificial neural networks is critical for further improving the performance of meta-predictor. A new pipeline based on this integration for miRNA target prediction has been developed. A strategy using outputs of individual predictors to reorganize large-scale miRNA:mRNA interaction dataset has also been validated and used to evaluate the prediction accuracy of predictors. The predictor is available at: https://github.com/xueLab/mirTarDANN).

**Electronic supplementary material:**

The online version of this article (10.1186/s12864-019-5528-1) contains supplementary material, which is available to authorized users.

## Background

MiRNAs are short RNA molecules of about 22 nucleotides [1], which are normally produced from non-coding RNAs by a process of two-step cleavage catalyzed by Drosha [2] and Dicer [3, 4], respectively. Although being very short compared to many other types of RNA molecules, miRNAs play critical roles in genome-wide gene expression regulation. Most miRNAs perform their functions through the well-known canonical pathway [5–7], in which an miRNA forms base pairing with the 3′-UTRs of its target mRNAs [8] to repress the expression of those mRNAs by either inhibiting translation or inducing mRNA degradation [5, 9]. Since the discovery of the first miRNA lin-4 in the 1990s [10], more and more miRNAs have been identified. Many mammal genomes each contain more than 2000 miRNAs [11]. These miRNAs were estimated to regulate about 60% of all genes in each genome [12–14].

Clearly, identifying the targets of miRNAs is critical for deciphering the functions of miRNAs, as well as revealing the mechanisms of gene expression regulation. For this purpose, many computational tools have been developed to predict the interaction partners of miRNAs [12, 15–30]. These tools are generally developed based on two categories of strategies: (I) base pairing between the seed region of miRNA and the 3′-UTR of mRNA, in integration with various sequential, structural, interaction, and evolutionary information [12, 22–26]; (II) machine learning based methods, such as hidden Markov Model (HMM) [16], support vector machine (SVM) [27, 28], and regression models [29]. With the application of these advanced techniques, significant progress has been achieved in the development of miRNA target predictors. Nonetheless, these predictors start to face many other challenges nowadays. Many predictors were trained and tested in rather small datasets when the predictors were developed. However, the amount of genomics/proteomics data is increasing very fast. Consequently, the accuracy of existing computational tools on newly emerged large-scale datasets becomes a question. In addition, the prediction accuracy of many existing miRNA target predictors is still not sufficient for direct applications [31]. Especially, the sensitivity of many existing predictors is rather low. Furthermore, when using machine learning based techniques to build a predictor, the common procedure is to build a dataset, select features, and then optimize the predictor. Clearly, when the dataset or the number of features used in the predictor becomes larger, the intrinsic noise associated with the dataset or the features will increase and may eventually impede the optimization of new predictors in large datasets. Therefore, it is also critical to develop new strategies to improve the prediction performance of predictors in large datasets.

In our previous studies on the development of various predictors [32–36], as well as the studies of other groups [30, 37, 38], individual predictors were integrated to make meta-prediction to improve the final prediction accuracy. However, a direct integration of the outputs of individual predictors may not improve the prediction accuracy significantly [32–34]. Under this situation, further integration of other techniques, such as non-linear transformation [34, 39] and dual-threshold value [35, 36], was very effective in improving prediction accuracy. In addition to using meta-strategy, splitting a large dataset into multiple smaller datasets is an alternative strategy for developing dataset-specific predictors and for combining dataset-specific predictors to improve the final prediction accuracy [35, 36]. In our previous work, dual-threshold and sequential voting were used to make meta-prediction for miRNA:mRNA interactions [35]. Nonetheless, the sample size is still insufficient, and the meta-predictor only integrates three individual predictors, including: miRanda, miRDB, and PITA [35]. Besides, it is also clear to us that dual-threshold sequential-voting has profound dependence on threshold values. Stringent threshold values will result in high-confidence predictions, but less number of true predictions. On the other hand, less-stringent threshold values will lead to a higher number of true predictions, but also low-confidence. Consequently, it is challenging to keep balance between confidence and number of true predictions, and therefore the effectiveness of dual-threshold sequential-voting is still limited. To solve these problems, we designed an upgraded strategy in this project focusing on the following aspects: (1) increasing sample size; (2) changing dual-threshold sequential-voting to dual-threshold two-step significance-voting. Here, dual-threshold still means that true prediction and false prediction have different threshold values. Two-step voting indicates there are two separate steps, in which the first step uses more stringent threshold values to generate high-confidence predictions, while the second step uses less-stringent threshold values to create extra true predictions. Significance voting allows the comparison of distance from predictive scores to threshold values between different individual predictors; and (3) integrating an artificial neural network (ANN) into each branch of the decision tree. The reason for this integration is that the outcomes of decision tree discussed in [2] are either high-confidence, or low-confidence. Therefore, ANN can be used here to refine the prediction.

## Results

### Performance of individual predictors in eleven predictor-specific datasets

A large dataset containing more than 40,000 miRNA:mRNA interactions was assembled. Many samples in this dataset do not have numerical outputs from the following four miRNA target predictors: MiRanda [24], MiRDB [28], PITA [17], and TargetScan [12]. Therefore, for effectively comparing the prediction performance of predictors and integrating outputs of individual predictors into meta-predictor, all the samples in the dataset were re-organized into subsets based on the availability of numerical outputs of these individual predictors. Consequently, one D4, four D3 (D3–1, D3–2, D3–3, and D3–4), and six D2 (D2–1, …, D2–6) subsets were generated. Here, “D” stands for dataset, the number following “D” shows the number of individual predictors associated with that subset, the numbers after dash represent various combinations of individual predictors (please see Method for a more detailed description of these subsets). Clearly, the performance of four individual predictors can be compared in each of these subsets.

**Fig. 1:**
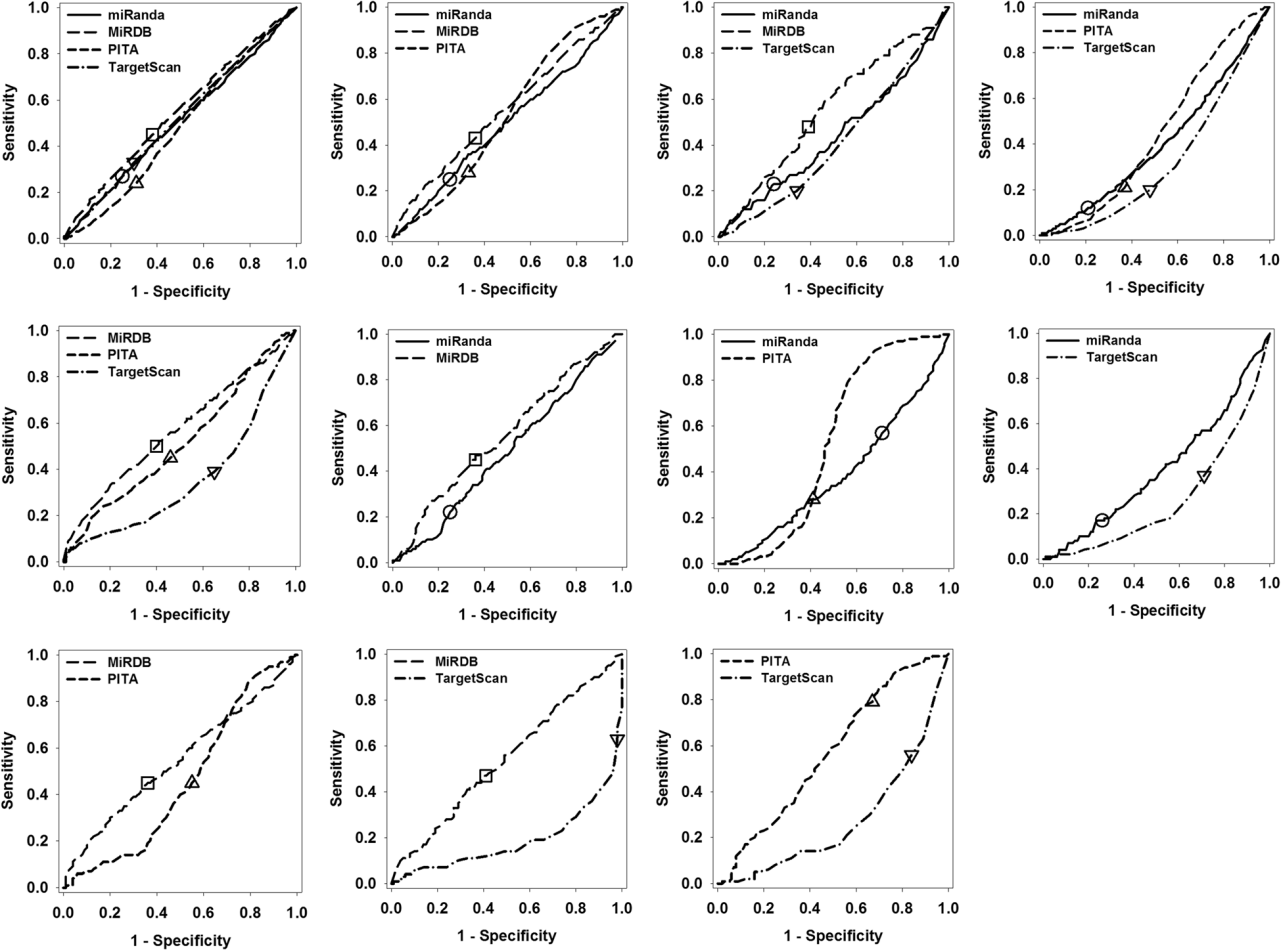
ROC curves of individual predictors in the eleven newly designed non-redundant datasets. The datasets from top to bottom and from left to right are: D4, D3–1, D3–2, D3–3, D3–4, D2–1, D2–2, D2–3, D2–4, D2–5, and D2–6, respectively. Samples in the D4 dataset has prediction scores from four individual predictors, therefore, there are four ROC curves each for a predictor. Similarly, the D3 series datasets and D2 series datasets have three and two ROC curves, respectively. In each of the insets, x-axis shows the value of 1-specificity, while y-axis shows the values of sensitivity

Figure 1 shows the Receiver Operating Characteristic (ROC) curves of individual predictors in the eleven predictor-specific datasets. In overall, the prediction performance of four predictors in all the datasets is not optimal and still has a lot of room for improvement. In addition, in different datasets, different predictors may have large variations in prediction performance, as indicated by the shape of ROC curves, the sensitivity and specificity under their default threshold values. More specifically, in terms of AUC (Area Under the ROC Curve), miRDB achieved the highest value in the D4, D3–1, D3–2, D3–4, D2–1, D2–4, and D2–5 datasets, PITA was ranked at the first position in the D3–3, D2–2, and D2–6 datasets, and miRanda obtained the best result in the D2–3 dataset. While using sensitivity, miRDB is better than other predictors in the D4, D3–1, D3–2, D3–4, and D2–1 datasets, PITA outperforms other predictors in the D3–3, D2–4, and D2–6 datasets, TargetScan beats other predictors in the D2–3 and D2–5 datasets, and miRanda exceeds others in the D2–2 dataset. Clearly, using predictive scores of individual predictors to split a large dataset into multiple smaller subsets may provide a novel start point for evaluating the performance of different predictors. It shall also be noted that the datasets used in Fig. 1 do not contain redundant samples. By taking into consideration that predictive scores of a predictor may distribute in a very narrow range, removing redundant samples may influence the calculated prediction accuracy significantly. The details are shown in Additional file 1: Figure S1 and will be discussed further in the discussion section.

### Combinations of techniques and prediction accuracy

As indicated by the above analysis, the performance of individual predictors in all subsets may have a large room for improvement. In this project, an integration strategy was designed for each subset as follows (please also see Method): First, dual-threshold value and two-step significance voting are combined to sort the outputs of individual predictors. Then, an ANN was used to integrate the outputs of individual predictors to make meta-prediction. It should be noted that dual-threshold value and two-step significance-voting are used to determine the infrastructure of decision-tree. They are not used separately in decision tree in this project for the following reasons: (1) only using dual-threshold values in meta-strategy showed many limitations in our previous project [35]; and (2) using two-step significance-voting alone in meta-predictor causes a lot of issues on the selection of threshold values. Therefore, these two techniques are essentially a single component of the designed meta-strategy. With that said, there are actually two categories of techniques in the meta-predictor: decision tree (incl. Dual-threshold and two-step significance-voting) and ANN. Consequently, there are three different combinations of these techniques: (1) individual predictors + decision tree (C-I); (2) individual predictors + ANN (C-II); and (3) individual predictors + decision tree + ANN (mirTarDANN).

The prediction performance of these three combinations in all eleven subsets under multi-fold cross-validation is presented in Table 1. Clearly, the first combination has only limited efficacy in improving prediction performance. There is always large imbalance between sensitivity and specificity. The second combination may have significantly improved accuracy (ACC) in several subsets, such as D3–1 and D3–2, but very low sensitivity (Sens). The third combination showed significant improvement of prediction performance in most subsets under multiple accuracy measures. Therefore, the third combination will be used as the formal model of meta-predictor for further analysis. The third combination, MirTarDANN, has the following advantages: (1) higher accuracy in most datasets; (2) improved balance between sensitivity and specificity; (3) higher sensitivity in most datasets. The final model of MirTarDANN actually contains eleven modules, including one DANN-4, four DANN-3x, and six DANN-2x modules. Each of the modules is trained and validated in its corresponding subset, which is either D4 subset, or one of four D3-x subsets, or one of six D2-x subsets. Each subset was also split into multiple groups for multi-fold cross-validation and independent test.

**Table 1:** Prediction performance of three meta-predictors using three different sets of techniques

Dataset	C-I			C-II			mirTarDANN		
	Sens	Spec	Acc	Sens	Spec	Acc	Sens	Spec	Acc
D4	0.240 ± 0.120	**0.780 ± 0.128**	0.735 ± 0.108	0.357 ± 0.093	0.733 ± 0.087	0.702 ± 0.072	**0.568 ± 0.049**	0.555 ± 0.031	0.556 ± 0.025
D3–1	0.475 ± 0.063	0.602 ± 0.036	0.584 ± 0.022	0.297 ± 0.032	**0.789 ± 0.044**	**0.716 ± 0.031**	**0.602 ± 0.048**	0.530 ± 0.041	0.541 ± 0.032
D3–2	**0.671 ± 0.097**	0.235 ± 0.108	0.268 ± 0.094	0.433 ± 0.128	**0.690 ± 0.041**	**0.669 ± 0.036**	0.511 ± 0.176	0.605 ± 0.111	0.593 ± 0.092
D3–3	0.087 ± 0.018	0.693 ± 0.042	0.557 ± 0.028	**0.693 ± 0.052**	0.704 ± 0.069	0.701 ± 0.047	0.660 ± 0.037	**0.730 ± 0.056**	**0.715 ± 0.037**
D3–4	0.383 ± 0.058	0.434 ± 0.088	0.420 ± 0.050	0.622 ± 0.061	**0.752 ± 0.030**	**0.713 ± 0.012**	**0.650 ± 0.051**	0.722 ± 0.051	0.700 ± 0.026
D2–1	**0.700 ± 0.073**	0.221 ± 0.040	0.334 ± 0.029	0.402 ± 0.065	0.653 ± 0.040	0.593 ± 0.013	0.312 ± 0.066	**0.722 ± 0.078**	**0.624 ± 0.037**
D2–2	0.519 ± 0.049	0.444 ± 0.040	0.480 ± 0.023	0.668 ± 0.202	0.728 ± 0.017	0.701 ± 0.094	**0.685 ± 0.102**	**0.796 ± 0.020**	**0.745 ± 0.038**
D2–3	0.306 ± 0.137	0.307 ± 0.062	0.312 ± 0.035	0.527 ± 0.055	**0.849 ± 0.019**	0.738 ± 0.042	**0.558 ± 0.047**	**0.849 ± 0.026**	**0.749 ± 0.017**
D2–4	0.398 ± 0.024	**0.659 ± 0.038**	0.509 ± 0.004	**0.777 ± 0.081**	0.393 ± 0.182	0.608 ± 0.050	0.745 ± 0.023	0.540 ± 0.029	**0.656 ± 0.023**
D2–5	0.388 ± 0.175	0.188 ± 0.174	0.276 ± 0.044	0.700 ± 0.079	0.837 ± 0.060	0.779 ± 0.011	**0.721 ± 0.086**	**0.849 ± 0.022**	**0.797 ± 0.050**
D2–6	**0.700 ± 0.155**	0.255 ± 0.032	0.466 ± 0.063	0.678 ± 0.065	0.724 ± 0.048	0.700 ± 0.038	0.622 ± 0.074	**0.769 ± 0.060**	0.693 ± 0.017

C-I integrates individual predictors with a decision tree, C-II uses ANNs to integrate individual predictors, and mirTarDANN is a combination of individual predictors, decision tree, and ANNs. The performance is measured by sensitivity (Sens), specificity (Spec), and accuracy (Acc) under multi-fold cross-validation. The high-lighted values are the highest among three meta-predictors in the same subset

### Performance of new predictor mirTarDANN

**Fig. 2:**
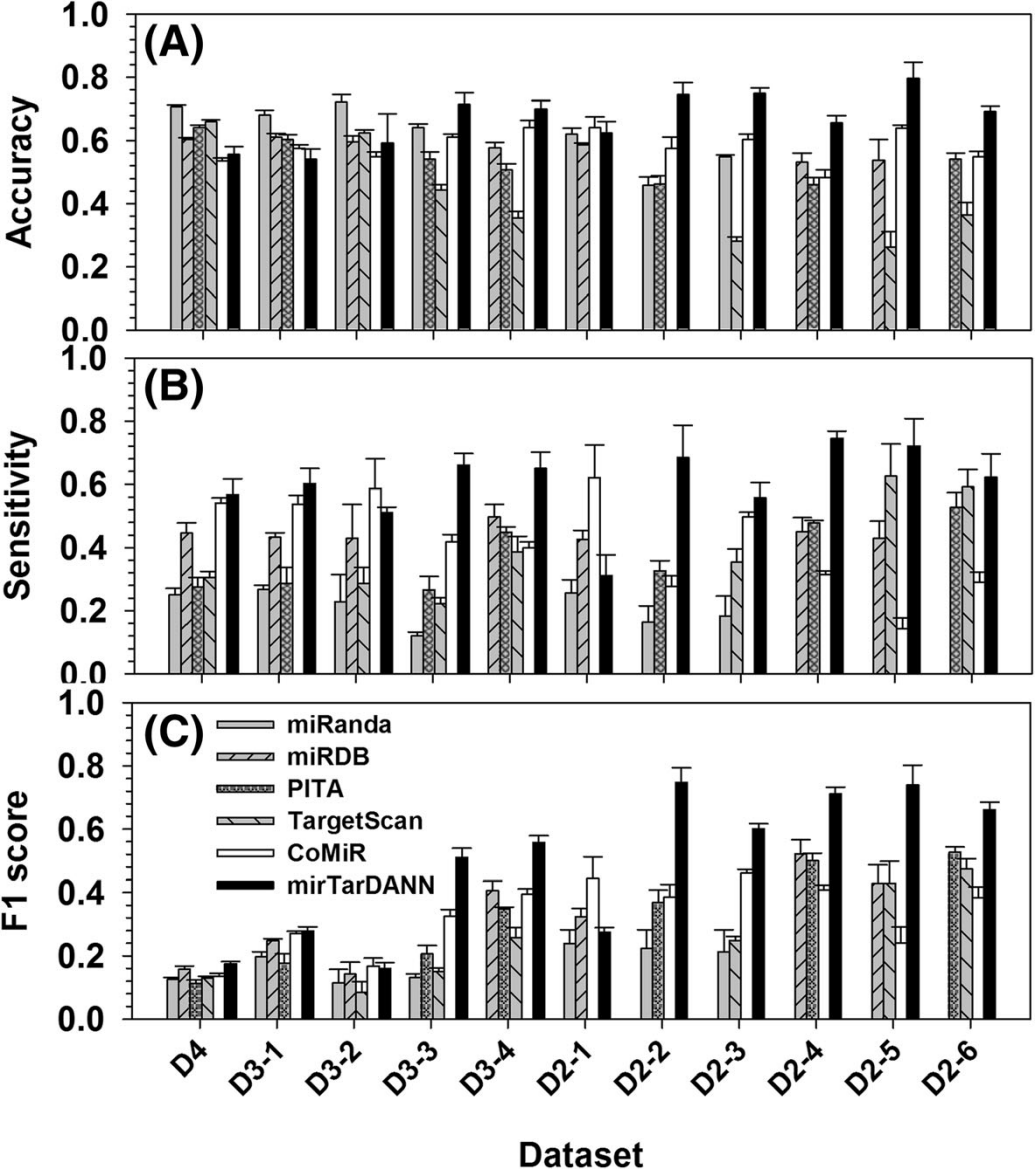
Comparison of prediction performance of different predictors in eleven newly-designed datasets under multi-fold cross validation. X-axis shows the eleven newly-designed datasets, while the y-axis shows (**a**) accuracy, (**b**) sensitivity, and (**c**) F1 score, respectively. Error bars are standard deviation from multi-fold cross validation. In the D4 dataset, the performance of mirTarDANN was compared with four individual predictors and ComiR. In each of the D3 series datasets, mirTarDANN was compared to ComiR, and three out of four individual predictors. In each of the D2 series datasets, mirTarDANN was compared to ComiR and two out of four individual predictors. When calculating the accuracy of individual predictors, their default cutoff values were used. For ComiR, a false discovery rate of 5% was recommended by the developer to determine the cutoff. Therefore, based on the calculations of 50 randomly selected miRNAs and their targets in the datasets, 0.82 was used as the cutoff of ComiR

Figure 2 shows the comparison of accuracy, sensitivity, and F1 score under multi-fold cross validation among miRanda, miRDB, PITA, TargetScan, ComiR, and the newly designed meta-predictor mirTarDANN. Clearly, in terms of accuracy, mirTarDANN outperformed other predictors in the D3–3, D3–4, D2–2, D2–3, D2–4, D2–5, and D2–6 datasets, matched to others in the D2–1 dataset, but fell behind other predictors in the D4, D3–1, and D3–2 datasets. In terms of sensitivity, mirTarDANN exceeded other predictors in almost all the datasets except D3–2 and D2–1. In these two datasets, four individual predictors have low sensitivity but high accuracy due to having high specificity (see Additional file 1: Table S2). This is also supported by the fact that the sensitivity of mirTarDANN is higher than individual predictors in these two datasets. Anyhow, in these two datasets, ComiR achieved reasonably high or the highest values on both sensitivity and accuracy. In terms of F1 score, mirTarDANN achieved significantly improved F1 score in D3–3, D3–4, and another five D2 datasets (except D2–1), and equal or comparable scores with ComiR and/or other individual predictors in the D4, D3–1, and D3–2 datasets. In D2–1 dataset, ComiR’s F1 score beat all other predictors, including mirTarDANN. The overall accuracy and sensitivity of mirTarDANN averaged in all the eleven datasets under multi-fold cross validation is 59.5 and 59.0%, which are 3 and 9% higher than ComiR, the predictor at the second position. The F1 and MCC values of mirTarDANN as an average of all the eleven datasets are 0.492 and 0.287, compared to 0.329 and 0.101 from ComiR, respectively (see Additional file 1: Table S2 & S3).

**Fig. 3:**
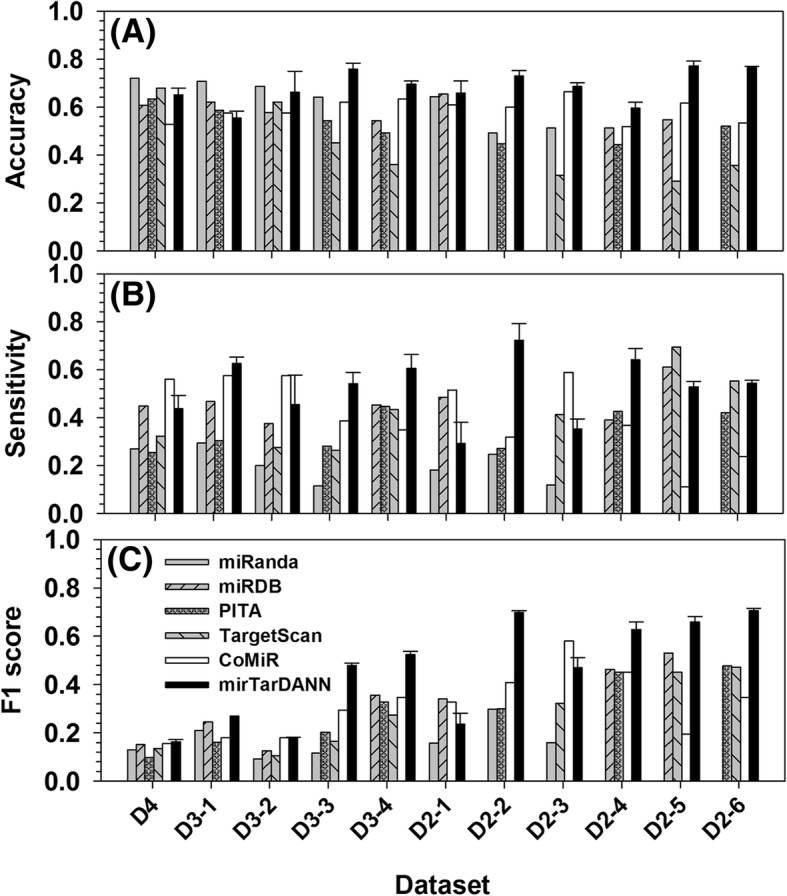
Comparison of prediction performance of different predictors in the independent datasets that each is associated with one of the eleven newly-designed datasets. X-axis shows the datasets, while the y-axis shows (**a**) accuracy, (**b**) sensitivity, and (**c**) F1 score, respectively. Only mirTarDANN has error bars since mirTarDANN has multiple sets of parameters optimized under multi-fold cross-validation

Figure 3 shows the comparison of accuracy, sensitivity, and F1 score among four individual predictors, ComiR, and mirTarDANN in the independent test datasets. The overall trend of data in this figure is similar to those in Fig. 2. Basically, mirTarDANN has the highest accuracy in all datasets except D4, D3–1, and D3–2. In terms of sensitivity, mirTarDANN outperformed other predictors significantly in D3–1, D3–3, D3–4, D2–2, and D2–4 datasets, fell behind ComiR in the D4, D3–2, D2–1, and D2–3 datasets, and also fell behind one or two individual predictors in the D2–1, D2–5, and D2–6 datasets. As to F1 score, mirTarDANN was only behind ComiR in the D2–1 and D2–3 datasets. As an average in all the eleven test datasets, the accuracy of mirTarDANN and ComiR is 65.1% vs 56.2%, and the sensitivity of mirTarDANN and ComiR is 50.6% vs 51.4%, the F1 scores are 0.455 (miRTarDANN) vs 0.315 (ComiR), and the MCC values are 0.276 (mirTarDANN) vs 0.119 (ComiR) (see Additional file 1: Table S4 and S5).

### Cross-subset performance of the newly-designed meta-strategy/meta-predictor

**Fig. 4:**
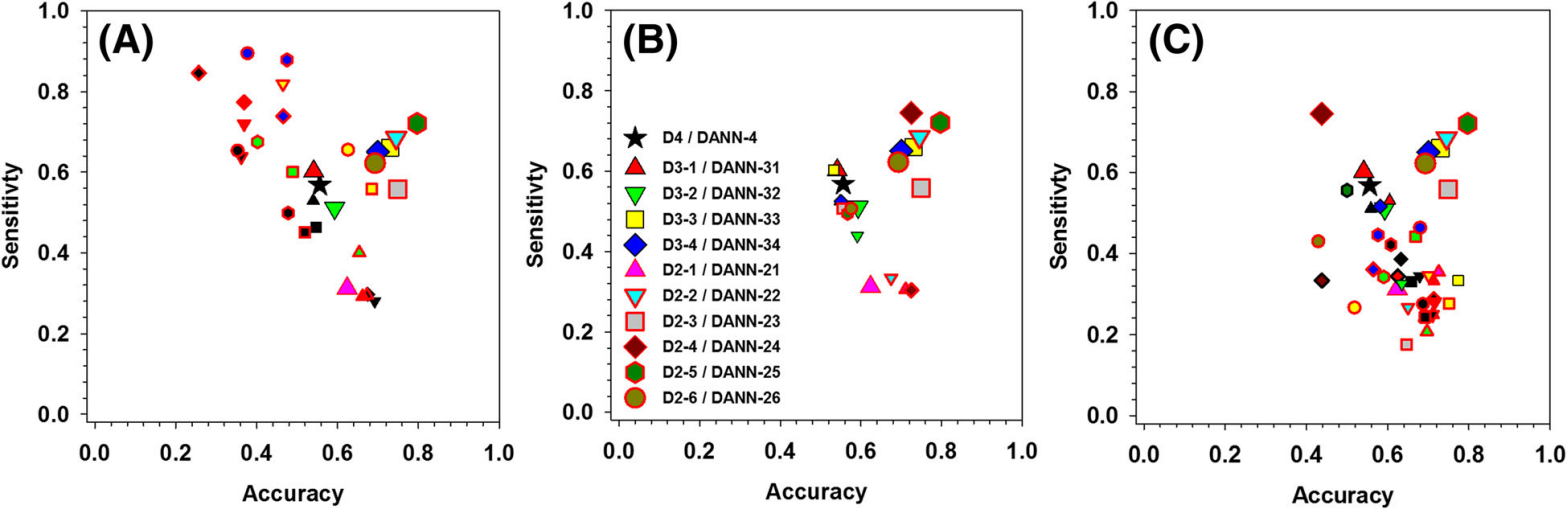
Performance of eleven DANN modules in different subsets. **a**Performance of original trained DANN modules in different subsets. **b** Performance of DANN modules re-trained in merged subsets. **c** Performance of re-trained DANN modules in original subsets. X-axis shows the prediction accuracy, while y-axis shows the sensitivity. Each symbol stands for the performance of a specific subset predicted by specific DANN module. Larger symbols are performance of eleven original DANN modules trained in their corresponding subsets, while small symbols show performance of original DANN modules in non-corresponding subsets or performance of re-trained DANN modules in various subsets. The subsets are represented by the colours filled in the symbols as follows: D4:black, D3–1:red, D3–2:green, D3–3:yellow, D3–4:blue, D2–1:pink, D2–2:cyan, D2–3:grey, D2–4:dark red, D2–5:dark green, D2–6:dar yellow. DANN modules are denoted by the shape of symbols as follows (All DANN-3x have dark edges, while all DANN-2x have red edges): DANN4:star, DANN-31:up triangle, DANN-32:down triangle, DANN-33:square, DANN-34:diamond, DANN-21:up triangle, DANN-22:down triangle, DANN-23:square, DANN-24:diamond, DANN-25:hex, DANN-26:circle

The meta-predictor is composed of eleven modules, and each module is associated with a specific set of individual predictors. Each module is also trained and validated in its corresponding subset, in which all the samples have and only have numerical predictive scores from that set of individual predictors. Apparently, modules trained in subsets with less number of individual predictors may be used to make prediction for samples with more predictive scores. For example, DANN-21 can be used to make prediction for samples in the D4 and D3–1 subsets. In other words, D4 samples can be predicted by not only DANN-4 module, but also four DANN-3x modules and six DANN-2x modules. Samples in D3–1 subset have miRanda, miRDB, and PITA scores, therefore, D3–1 samples can also be predicted by DANN-21 (requires miRanda and miRDB scores), DANN-22 (requires miRanda and PITA scores), and DANN-24 (requires miRDB and PITA scores) modules. In this project, the prediction accuracy of a trained DANN module in a non-corresponding subset is called cross-subset accuracy. Figure 4 shows the performance of DANN modules in different subsets. In this figure, x-axis is the prediction accuracy and y-axis shows the sensitivity. Therefore, when a DANN module has good performance in a subset, the symbol shall be at the upper-right corner along the diagonal line. Clearly, as shown in Fig. 4(a), all the DANN modules in mirTarDANN have lower performance in cross-subset test.

It is expected that DANN modules have lower performance in cross-subset validation, since a specific DANN module has been trained in its corresponding subset, not the other subsets used for cross-subset validation. To further evaluate the influence of different subsets on the performance of specific modules, all the associated subsets were merged to make a merged-subset and the corresponding DANN module was re-trained in the merged-subset to see the final prediction accuracy under multi-fold cross-validation. Let’s take D4 subset as an example as follows: all the D4 samples can be predicted by DANN-4 and DANN-31 modules. However, since DANN-31 was trained in the D3–1 subset, it has lower prediction performance in D4 dataset. Therefore, it becomes interesting to see if D4 and D3–1 can be merged to make a new D3–1 subset (i.e. *D3–1) to train a new DANN-31 module (i.e. *DANN-31) to improve the prediction performance in D4, D3–1, or *D3–1. In this case, for the purpose to re-train DANN-31 module, D4 and D3–1 become associated datasets. In this project, only the merge of a subset associated with more individual predictors into a subset associated with less individual predictors is analysed, merge in the reversed direction will result in samples with non-numerical predictive scores and therefore is not further considered. Clearly, the similar analysis can be performed for all the DANN-3x and DANN-2x modules. The results are shown in Fig. 4(b) and (c), accordingly. In Fig. 4(b), the performance of each re-trained DANN module in its corresponding merged-subset was analysed. In Fig. 4(c), the performance of each re-trained DANN in its original associated subsets was analysed. Clearly, even if a new DANN module is re-trained in the corresponding merged-subset, the performance of this re-trained module is still less satisfactory in the merged-subset, as well as in the original associated subsets.

### miRNA:mRNA interactions exclusively identified by mirTarDANN are closely associated with diseases

**Fig. 5:**
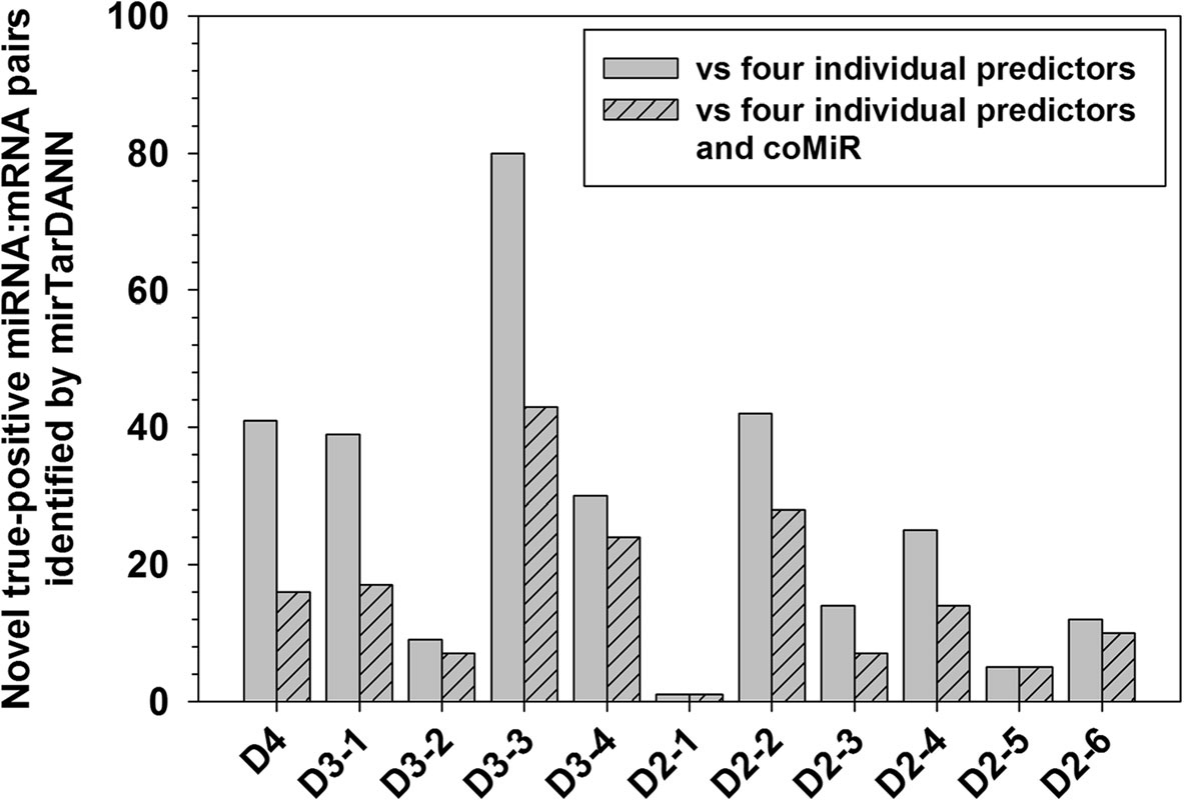
Number of novel miRNA:mRNA interactions identified by mirTarDANN in the eleven newly-designed datasets. Grey bars shows the number of novel interactions compared to four individual predictors, including miRanda, miRDB, PITA, and TargetScan. Dashed grey bars show the difference between mirTarDANN and one of the other five predictors, including four individual predictors and ComiR

In all the eleven datasets, the newly designed meta-predictor mirTarDANN exclusively identified 298 experimentally validated miRNA:mRNA interactions that cannot be identified by any of the individual predictors under their default settings as shown in Fig. 5. Out of these 298 miRNA:mRNA interactions, 126 can be predicted by ComiR. Therefore, mirTarDANN identified 172 novel miRNA:mRNA interactions that can not be identified by four individual predictors and ComiR (see Fig. 5).

Among these 172 novel true positive predictions, many are from the same dataset. For example, 43, 28, 24, 17, 16, and 14 interactions are from D3–3, D2–2, D3–4, D3–1, D4, and D2–4 datasets. The other thirty true positive predictions are from the other five datasets, with each dataset contributing one to ten true-positive predictions. These miRNA:mRNA interactions involve 167 mRNAs and 82 miRNAs.

Among the afore-mentioned 167 mRNAs, ACO1, SMARCA2, SUMF1, TOX4, and ZMAT3 can be regulated by more than one miRNAs, multiple mRNAs belong to the same gene families, such as members in the AKAP, EIF, MED, SLC, TMEM, USP, ZBTB, ZFP, and ZMAT families. ACO1 is a cytoplasmic aconitate hydratase. When cellular iron levels are high, ACO1 binds to iron and serves as an aconitase to generate the isocitrate during the citric acid cycle [40]. When iron levels are low, ACO1 binds to iron-responsive elements (IRES) in target mRNA molecules to serve as a RNA-binding protein [41]. In the predicted novel miRNA:mRNA interactions, ACO1 is regulated by MMU-MIR-339 and MMU-MIR-10B. SMARCA2 has the function of ATP-dependent helicase and is probably a global transcription activator. It is a component of SWI/SNF chromatin remodelling complexes [42, 43]. It is also a component of npBAF complex, and therefore critical for the development of neural stem cells [44]. SMARCA2 is bound by MMU-MIR-33 and MMU-MIR-466A. SUMF1 is a formylglycine-generating enzyme [45, 46]. Its malfunction is the cause of multiple sulfatase deficiency (MSD) [47, 48]. SUMF1 is targeted by MMU-MIR-743B and MMU-MIR-488 in the predicted novel interactions. TOX4 (TOX high mobility group box family member 4) is a component of PTW/PP1 phosphotase complex, which regulates chromatin structures [49]. TOX4 is an interaction partner of MMU-MIR-221 and MMU-MIR-376C. ZMAT3 is the short name for Zinc finger matrin-type protein 3. It is a target of P53 and has functional roles in P-53 dependent regulatory pathway [50–52]. ZMAT3 is regulated by MMU-MIR-764 and MMU-MIR-292A.

**Fig. 6:**
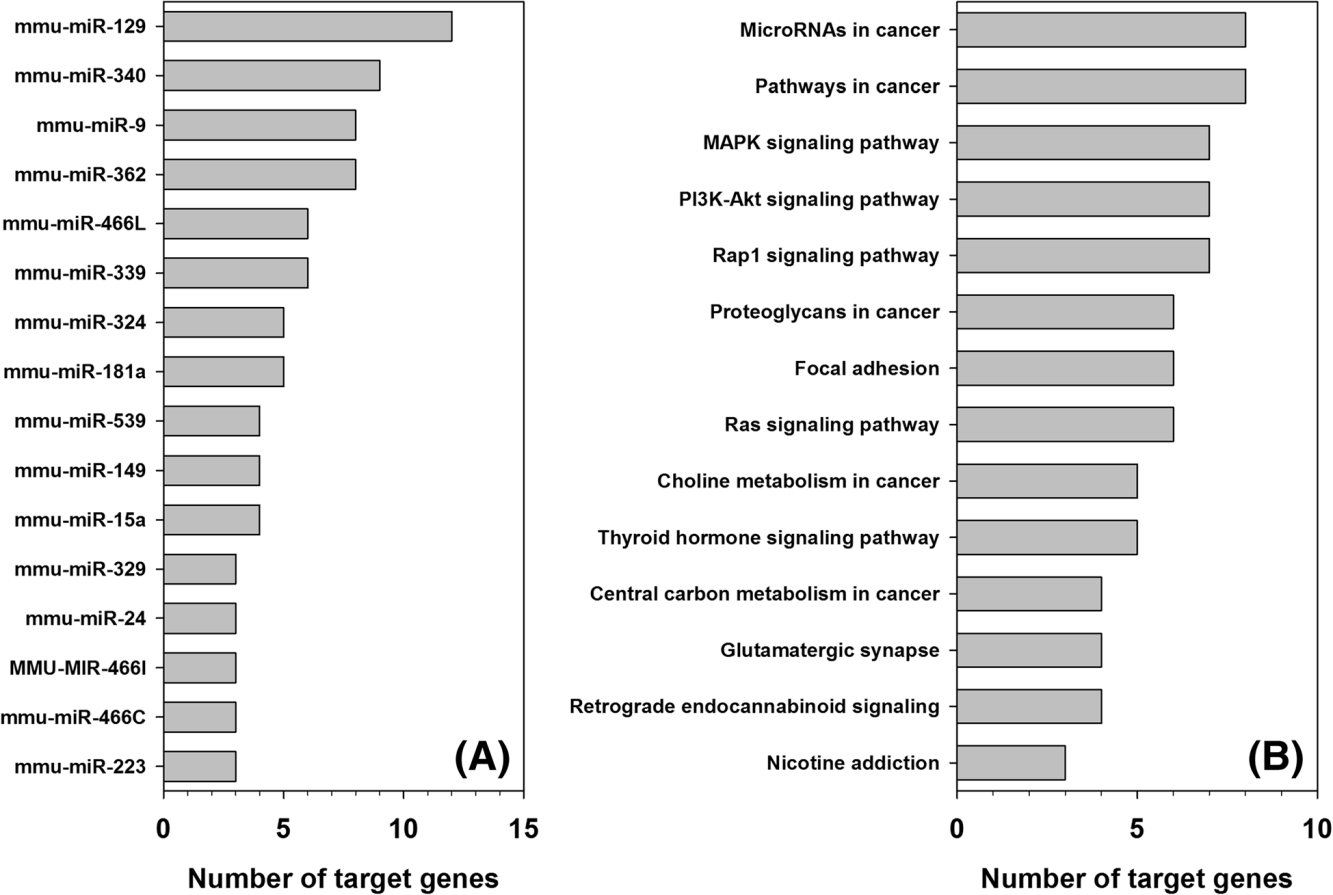
Functional analysis of novel miRNA:mRNA interactions identified mirTarDANN, but not identified by four individual predictors and ComiR.** a** List of miRNAs and the corresponding number of mRNAs that can be regulated by each of these miRNA. Only miRNAs having more than three mRNA targets are shown in the Fig. (**b**) KEGG pathways containing three or more genes, which are found in the novel miRNA:mRNA interactions

Out of the afore-mentioned 82 miRNAs, 36 may regulate multiple novel-predicted mRNAs. In which, MMU-MIR-129, MMU-MIR-340, MMU-MIR-362, and MMU-MIR-9 may regulate 12, 9, 8, and 8 mRNAs, respectively. These thirty-seven downstream mRNAs are involved in over thirty signalling pathways, including cancer-related pathways, MARK signalling pathway, Ras signalling pathway, hippo signalling pathway, RNA transport pathway, spliceosome pathway, etc. Especially, among those thirty-seven downstream genes, PDGFRA and MYLK are involved in eighteen and eight signalling pathways, respectively. In addition to the afore-mentioned four miRNAs, another 12 miRNAs each may regulate more than three novel-predicted mRNAs as shown in Fig. 6(a). The regulated signalling pathways and the number of involved genes of each pathways for the afore-mentioned 36 miRNAs are presented in Fig. 6(b).

## Discussion

Meta-strategy is capable to integrate the advantages of different individual predictors and therefore to improve the overall prediction accuracy efficiently. as well as balance sensitivity and specificity. Nonetheless, using a simple meta-strategy to directly integrate the outputs of individual predictors may not improve the prediction performance significantly [34, 35]. Therefore, it may be critical to integrate various data analysis techniques in meta-strategy to improve the final prediction accuracy. In this project, the predictive scores of miRanda, miRDB, PITA, and targetScan were input into a decision tree. In the decision tree, dual-threshold value, two-step significance-voting, and analysis of information-gain were integrated to filter the input scores of individual predictors. The input scores were then fed into an ANN to make meta-prediction. This meta-strategy has improved the prediction performance significantly, comparing to individual predictors and other meta-predictors. With the success of this strategy, it could be expected that integrating more individual predictors, and/or more data analysis techniques, is able to further improve the prediction accuracy. While this expectation is reasonable, it should be noted that the margin effect of adding extra individual predictors into meta-predictor could be very limited [32–34]. In addition, adding extra individual predictors may indicate that there will be more subsets and consequently the smaller size of subsets. By taking into consideration that multiple subsets in this project have only ~ 300–400 samples, there may be additional concerns for using more individual predictors in miRNA target meta-predictors. The data analysis techniques used in this project are essentially able to reduce noise in datasets and/or at different stages of prediction. Therefore, it can be expected that additional techniques may be used to further reduce noise, and consequently to improve prediction accuracy.

The reasons for choosing the afore-mentioned four individual predictors are multi-fold. First, these four individual predictors are well designed and have been broadly used in this field. Second, these individual predictors have standalone versions, webservers, as well as pre-assembled prediction of miRNA:mRNA interactions for the entire mouse genome. Consequently, the comparison and application of our newly-designed meta-predictor becomes much easier. Third, the use of these four individual predictors is able to split the original large dataset into multiple smaller subsets, which each has reasonable amount of samples and well reduced noise as indicated by improved prediction performance. The original unsplit dataset used in this project contains more than 7000 positive miRNA:mRNA interactions and ~ 40,000 negative miRNA:mRNA interactions. Many samples in this dataset have non-numerical values, such as “-”, “NA”, or null, in the outputs of those four individual predictors. Under the current scheme of the newly-designed meta-strategy, these non-numerical values cannot be used directly in the decision tree. Therefore, it is necessary to organize all the samples into multiple subsets based on the availability of numerical scores of individual predictors. In this way, all the samples in a subset have and only have numerical values from a specific subset of individual predictors. For example, all the samples in the D3–1 subset have and only have numerical predictive values from miRanda, miRDB, and PITA. Consequently, these scores can be compared to threshold values associated with the DANN-31 module of the decision tree and fed into the ANN associated with DANN-31 to make refined prediction. The second advantage of splitting a large dataset into multiple smaller subsets is to reduce the intrinsic noise of the subsets. Normally, a sample with a non-numerical value can only be assigned as false, even the sample is a positive sample. This sample is also a sample space, different from other samples with numerical values. Therefore, the presence of samples with non-numerical values increases the noise of datasets. Another advantage of using subsets is that the prediction accuracy of predictors can be measured more specifically in these subsets, and thus the comparison of prediction accuracy of different predictors in subsets is more informative. Actually, as shown in Figure 1, the performance of individual predictors has large variations in different subsets, and the variation can only be captured in the form of subset. Furthermore, comparison of various accuracy measures in different subsets of different predictors provides another practical strategy to select the most appropriate predictors. For example, if a miRNA:mRNA pair has miRanda, miRDB, and TargetScan scores (or in other words, this pair is similar to samples in the D3–2 dataset), ComiR can be selected to make prediction to ensure the highest sensitivity, while miRanda could be used to ensure a higher specificity.

**Fig. 7:**
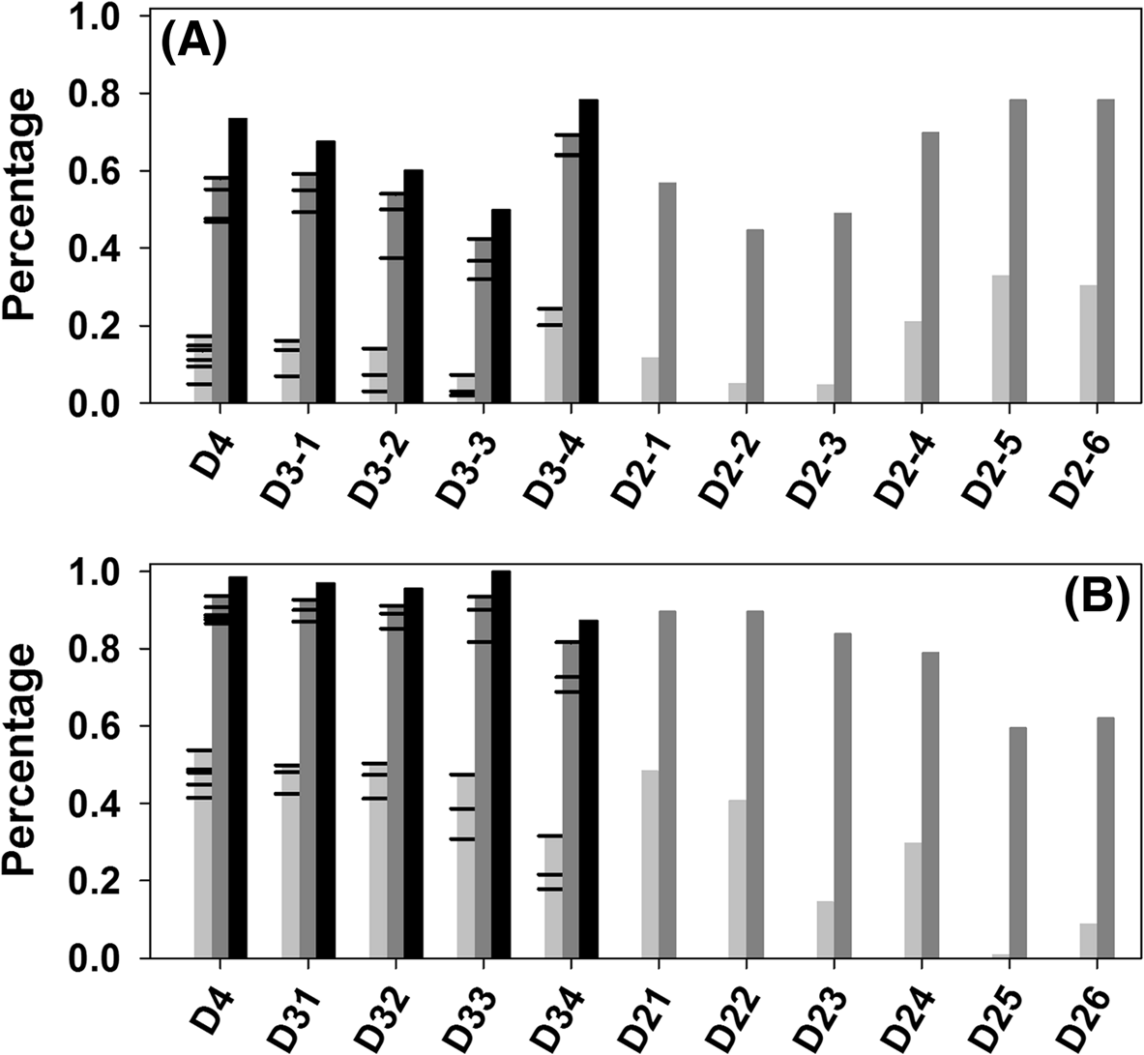
Overlap and coverage between individual predictors for (**a**) positive samples and (**b**) negative samples. Grey bars are the values of overlap between two predictors (pairwise overlap). Each cap indicates the value of overlap for a specific pair of predictors. Dark grey bars stand for the values of coverage between two predictors (pairwise coverage), with caps each for a specific pair of predictors. Black bars show values of all-inclusive coverage, which are calculated from all predictors in that dataset. Apparently, only D4 and D3 series datasets have the all-inclusive coverage

The reason for using those four individual predictors can also be rationalized in the analysis of overlap and coverage. The overlap of two predictors refers to the numbers of overlapped true positive and overlapped true negative predictions, the coverage of two predictors shows the maximum number of non-overlapped true positive or true negative predictions of two predictors. Clearly, overlap is an indicator of the similarity between two predictors, while coverage shows the maximum number of correct predictions that can be made by these two predictors. For the simplicity of comparison, both overlap and coverage are shown in percentile by dividing the total number of either positive or negative samples in that dataset. Figure 7(a) shows the values of overlap and coverage of different pairs of predictors for positive samples in the eleven datasets. Clearly, the values of pairwise overlap are in most cases around or below 20% except in the following three datasets: D3–4, D2–5, and D2–6, where the values of positive sample overlap are still less than 30–40%. In terms of pairwise coverage, the values normally reach to ~ 40% in the D3–3, D2–2, and D2–3 datasets, to ~ 60% in the D4, D3–1, D3–2, and D2–1 datasets, and to ~ 70% in the D3–4, D2–4, D2–5, and D2–6 datasets. The maximum values of coverage of multiple predictors in the D4 and D3 series datasets are normally ~ 10% higher than the highest pairwise coverage in that dataset. The overlap and coverage of negative samples are presented in Fig. 7(b). In the D4, D3–2, D3–2, D3–3, D2–1, and D2–2 datasets, the pairwise overlap for negative samples are normally between 40 and 50%, and the pairwise coverage values are around ~ 90%. In the D3–4, D2–2, D2–3, and D2–4 datasets, the values of pairwise overlap are between 10 and 30%, and the values of pairwise coverage are normally around 80%. In the D2–5 and D2–5 datasets, the overlap is below 10% and the coverage is at ~ 60%. In the datasets involving three or four predictors, the maximum values of coverage of multiple predictors are normally 5% higher than the highest pairwise coverage values. Clearly, combining different predictors may improve the prediction results significantly due to the fact that the pairwise coverage values are much higher than the prediction accuracy of individual predictors.

The analysis of overlap and coverage demonstrates that these four predictors have some common predictions, but also many predictor-specific predictions. Therefore, the analysis of overlap and coverage is useful in determining the infrastructure of decision tree. For majority-voting based strategy, overlap is a critical measurement. However, in significance-voting based predictor[s], although overlap is still very important, coverage plays a more critical role. In addition, majority-voting is strong in selecting part of the true-positive predictions that have very high levels of confidence, significance-voting is able to pick up additional true-positive predictions that cannot be identified by majority-voting. In this way, significance-voting improves the overall prediction accuracy and has higher potential in application. To maximize the efficacy of significance-voting, the dual-threshold value and two-step voting strategies are also critical. In the first-step voting, the thresholds are normally stricter than those of single predictors. The purpose is to select high-confidence true-positive and true-negative predictions from individual predictors. The second step voting uses less-stringent threshold values to enable the selection of additional true-positive predictions that cannot be identified by individual predictors under their default settings.

After determining the infrastructure of the newly-designed meta-predictor, two sets of threshold values (the 1st-step threshold values for true positive and true negative, as well as the 2nd-step threshold values for true positive and true negative) need to be determined. Therefore, the information gain in each of the eleven datasets was analysed.

## Conclusions

Based on our previous studies where ANN or dual-threshold sequential-voting was used to integrate the predictive results of individual predictor, a novel infrastructure of meta-strategy that combines ANN and decision-tree was designed in this project to make meta-prediction of miRNA targets using predictive scores of four individual predictors, including miRanda, miRDB, PITA, and targetScan. Different from traditional decision-tree and the afore-mentioned dual-threshold sequential-voting, the new decision-tree uses dual-threshold and two-step significance voting. The combination of this new decision-tree with ANN was trained in a large-scale miRNA:mRNA interaction dataset and improved the prediction accuracy of miRNA:mRNA interactions significantly comparing to individual predictors and several other meta-predictors under multi-fold cross-validation and using independent datasets in multiple accuracy measures, such as accuracy, sensitivity, specificity, F1 score and MCC. Also, the availability of numerical scores of individual predictors was used to split a large dataset into multiple smaller subsets, to reduce the noise of subsets, improve the training of meta-predictor, and provide more specific evaluation of prediction performance of different predictors in the subsets. The reduce noise in the sunsets further improved the efficacy of meta-predictors developed on those subsets. The newly-designed meta-predictor identified near 200 novel miRNA:mRNA interactions that cannot be predicted by other predictors.

## Methods

### Datasets

MiRTarBase [53] and Tarbase [54] are very well designed large-scale miRNA:mRNA interaction databases. All the interactions in these two databases are validated by experimental techniques, such as reporter assay, western blot, Cross-Linking Immunoprecipitation (CLIP), and many others. The miRTarBase V7.0 contains nearly ~ 2000 mouse miRNAs, over 7000 target mouse genes, and 40,169 mutual interactions between them. The TarBase V7.0 contains more than 400 miRNAs, over 5000 mRNAs, and 28,923 miRNA:mRNA interactions. Although both containing a large amount of experimental data, these two databases have only 457 overlapped miRNA:mRNA interactions for the entire mouse genome. This fact gives grounds for combining these two databases in this study, as well as for developing novel computational strategies to predict miRNA:mRNA interactions.

MiRanda [24], MiRDB [28], PITA [17], and TargetScan [12] are four very popular predictors for miRNA:mRNA interactions. Their websites each provides a dataset containing the whole genome prediction of miRNA:mRNA interactions for mouse genome. For each possible miRNA:mRNA pair (or sample) in the genome, each predictor may produce either scored or unscored predictions. Only scored predictions were included in those datasets. There are over 810 k, 630 k, 2.7 m, and 270 k scored predictions for mouse genome, from miRanda, MiRDB, PITA, and TargetScan, respectively. Samples in these four datasets were further organized as follows: (I) Samples found in all four datasets were deposited into D4; (II) Samples found only in miRanda, MiRDB, and PITA, were saved in D3–1. Similarly, D3–2 for samples only found in miRanda, MiRDB, and TargetScan; D3–3 for samples only found in miRanda, PITA, and TargetScan; and D3–4 for samples common in MiRDB, PITA, and TargetScan; (III) Samples found only in miRanda and MiRDB were kept in D2–1. Similarly, there are another 5 subsets containing samples that are only found in two out of four datasets, which are D2–2 (MiRanda and PITA), D2–3 (MiRanda and TargetScan), D2–4 (MiRDB and PITA), D2–5 (MiRDB and TargetScan), and D2–6 (PITA and TargetScan); (IV) Samples found only in one dataset were grouped into another four datasets: D1–1 (miRanda), D1–2 (MiRDB), D1–3 (PITA), and D1–4 (TargetScan).

Then, samples in these subsets were compared with miRTarbase and TarBase. If a sample is found in either of these two databases, it is assigned as a positive sample, otherwise, negative sample. Afterwards, duplicated samples were identified if there was less than a 2% difference between all predictor scores of two samples. One of the duplicated samples was randomly selected and then removed. It should be noted here that since each D1 series dataset only contains samples with one prediction score, they were not further considered in this study. Consequently, there are in total eleven predictor-specific datasets, including one D4, four D3 series, and six D2 series datasets (Table 2).

**Table 2:** Numbers of samples in each of the eleven newly-designed datasets

Dataset	Associated individual predictors	No. of original samples	No. of non-redundant samples	No. of positive samples	No. of negative samples	No. of miRNAs	No. of mRNAs
D4	miRanda, MiRDB, PITA, TargetScan	45,517	22,446	1844	20,602	211	6747
D3–1	miRanda, MiRDB, PITA	29,486	9271	1339	7932	212	4619
D3–2	miRanda, MiRDB, TargetScan	7584	2478	198	2280	201	1097
D3–3	miRanda, PITA, TargetScan	107,813	5529	1220	4309	205	3541
D3–4	MiRDB, PITA, TargetScan	66,384	2984	864	2120	323	1946
D2–1	miRanda, MiRDB	5269	892	199	693	186	641
D2–2	miRanda, PITA	216,923	974	457	517	202	883
D2–3	miRanda, TargetScan	32,566	429	151	278	162	342
D2–4	MiRDB, PITA	29,531	810	455	355	165	645
D2–5	MiRDB, TargetScan	256,784	430	174	256	259	337
D2–6	PITA, TargetScan	384,944	363	179	184	175	288

### Meta-predictor

**Fig. 8:**
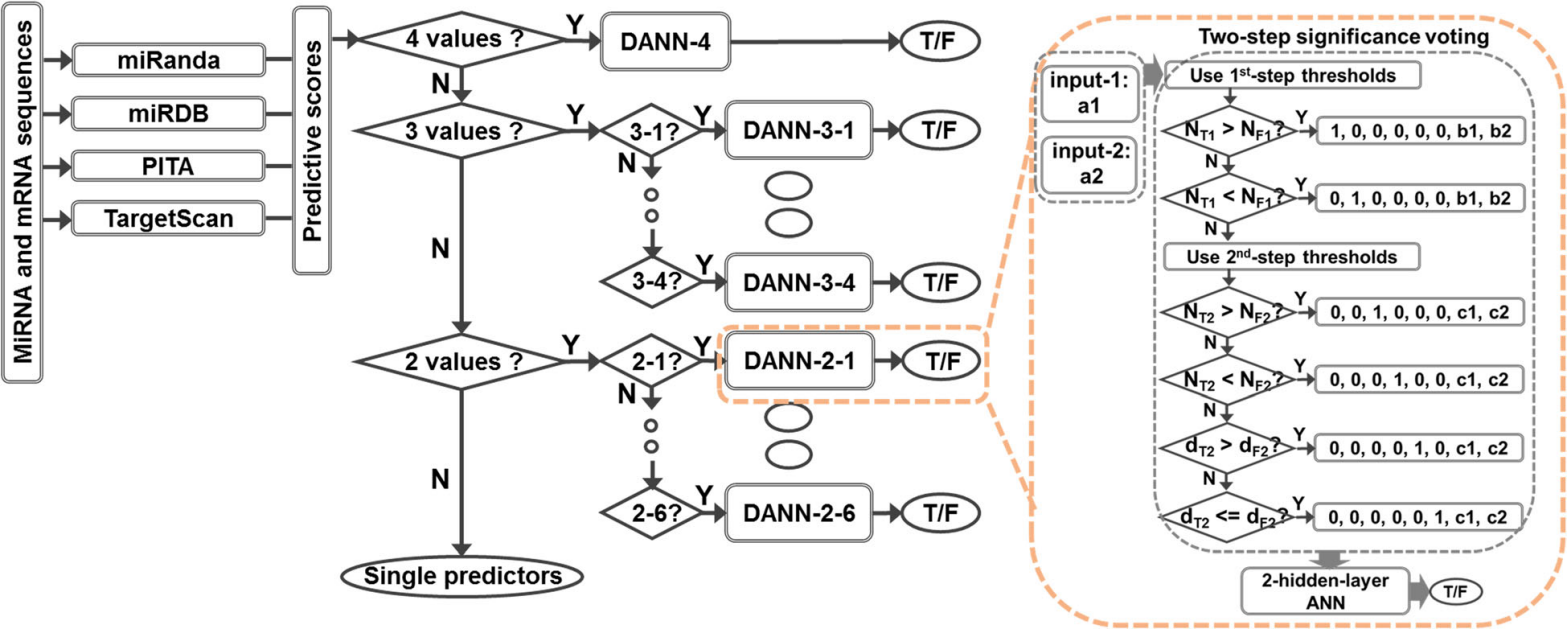
Infrastructure of decision-tree based meta-predictor. Query miRNA:mRNA sequences are firstly fed into miRanda, miRDB, PITA, and TargetScan to get individual predictions. These individual predictions may be scored or unscored (null output). Based on the scored individual predictions, a specific module of decision tree based artificial neural networks (DANN) will be selected. For example, if only miRanda and miRDB have scored predictions, module DANN-2-1 will be selected. There are eleven modules in the pipeline, each module corresponds to one of the eleven datatsets and uses scores different predictors as follows, DANN-4: miRanda, miRDB, PITA, and TargetScan; DANN-3-1: miRanda, miRDB, and PITA; DANN-3-2: miRanda, miRDB, and TargetScan; DANN-3-3: miRanda, PITA, and TargetScan; DANN-3-4: miRDB, PITA, and TargetScan; DANN-2-1: miRanda and miRDB; DANN-2-2: miRanda and PITA; DANN-2-3: miRanda and TargetScan; DANN-2-4: miRDB and PITA; DANN-2-5: miRDB and TargetScan; DANN-2-6: PITA and TargetScan. “Y” above an arrow and “N” along an arrow represent “Yes” and “No”. “T/F” inside a circle stands for true (T) or false (F) prediction. “N_T1_” is the number of predictors that make true prediction using the 1st-level true threshold values, and so on so forth for NF1, NT2, NF2. “b1” and “b2” are the differences of the predictions score from their corresponding 1st-level threshold values. “c1” and “c2” are the differences of the predictions score from their corresponding 2nd-level threshold values. “d_T2_” and “d_F2_” are the Euclidean distances of prediction scores from their corresponding 2nd-level threshold values for true (T) predictions and false (F) predictions, respectively. The infrastructure of the 2-hidden-layer ANN is described in the text. There are in total eleven DANNs

The infrastructure of the meta-predictor is shown in Fig. 8. For each pair of query miRNA and mRNA sequences, predictions are first made using four individual predictors including miRanda, MiRDB, PITA, and TargetScan. The predictive results are next examined to see if they are scored predictions. Based on the results of this examination, the predictive results are then input into a corresponding Decision-tree based Artificial Neural Network (DANN) to make the final meta-prediction. For example, if a query miRNA;mRNA sequence pair only has scored predictions from miRanda and miRDB, these two predictive scores will be fed into DANN-2-1, which uses the predictive results of miRanda and miRDB, and is trained in dataset D2–1.

Each DANN module uses the combination of several specific techniques including dual-threshold [35, 36], two-step significance-voting [36], and two-layer Artificial Neural Network (ANN). Dual-threshold means the threshold values of true and false predictions are different. Significance-voting refers to comparing the significance of predictive results by their Euclidean distances from corresponding threshold values. This technique is complementary to the majority-voting technique used in many studies. For example, when two predictors make true predictions and another two predictors make false predictions, comparing the number of true predictions (N_T_) and the number of false predictions (N_F_) may not lead to very useful conclusions. However, comparing the sum of distances from true thresholds value (d_T_) and the total distance from false threshold values (d_F_) may provide additional information of the relative significance of true predictions and false predictions. Two-step selection in this study uses two sets of dual-threshold values in combination with significance-voting as follows: First, the 1st-step threshold values, which are more stringent, are used to compare the number of true-prediction predictors and the number of false-prediction predictors. If the numbers are equal, the 2nd-step threshold values, which are less stringent, are used to compare the numbers of true-prediction predictors and false-prediction predictors. If the numbers are also equal, significance-voting based on the 2nd-step threshold values is applied. By using these techniques, the predictive results from individual predictors will be encoded in six different ways (see Fig. 8).

The encoded predictive results will be fed into one of the eleven DANNs, which is a fully-connected two-hidden-layer ANN. There are ten, twenty, and two nodes in the first hidden, second hidden, and output layers, respectively. Since there are two nodes in the output layer, the labels of positive and negative samples are [1,0] and [0,1], respectively. The number of nodes in the input layer is determined by the number of individual predictors in that DANN module. There are 10, 9, and 8 input nodes for DANN-4, DANN-3, and DANN-2 modules, accordingly. The activation function for all the nodes is hyperbolic tangent function. However, in the output layer, the output was further transformed using: $$ {O}_i^T=\raisebox{1ex}{${\mathit{\exp}}^{O_i}$}\!\left/ \!\raisebox{-1ex}{$\sum {\mathit{\exp}}^{O_i}$}\right.,i=1\  and\ 2 $$. In which, *O*_*i*_ and *O*^*T*^_*i*_ are the original output and transformed output, respectively.

### Training and validation

Each DANN predictor is trained and validated in one of the eleven predictor-specific datasets. For each of the datasets, 20% of all samples were randomly taken out to compose an independent test dataset, the other 80% of samples were randomly and equally split into multiple subsets for multi-fold cross-validation. Based on the number of samples in each dataset, either five-fold or three-fold cross-validation was used. The corresponding DANN was trained and validated using multi-fold cross validation in its corresponding predictor-specific dataset, as well as then validated in its independent test dataset.

### Preprocessing predictive results of individual predictors and information gain

The outputs of the afore-mentioned four individual predictors have different numerical ranges as follows: miRanda (− 1.364, − 0.1), miRDB (50, 100), PITA (− 43.24, 21.4), and TargetScan (− 9.05, 0). The default threshold values for true predictions of these predictors are <− 1.0, > 80, <− 10, and < − 0.36, respectively. For the simplicity of future data analysis, miRDB’s predictive scores are inversed by multiplying − 1, and all the scores from each individual predictor are scaled into the range (− 1,1). Thus, the corresponding true prediction threshold values for four predictors in their default settings are <− 0.424, <− 0.2, < 0.028, and < 0.920, accordingly.

Information Gain (IG) was calculated as a function of predictive score as follows:

$$ IG\left(\mathrm{x}\right)=\sum \limits_{i=1,2}{p}_i{\log}_2{p}_i-\sum \limits_{j=1,2}{f}_j(x)\ \sum \limits_{k=1,2}{p}_{j,k}\ {\log}_2{p}_{j,k} $$

In which, *p*_*i*_ is the fraction of positive (i = 1) or negative (i = 2) samples in the dataset. “x” is the threshold prediction score to split the dataset into two groups, f_j_(x) is the fraction of samples with prediction score higher than the threshold (j = 1) or the fraction of samples with prediction score lower than the threshold (j = 2), p_j,k_ refers to the fraction of positive samples (k = 1) or negative samples (k = 2) in the j-th group.

**Fig. 9:**
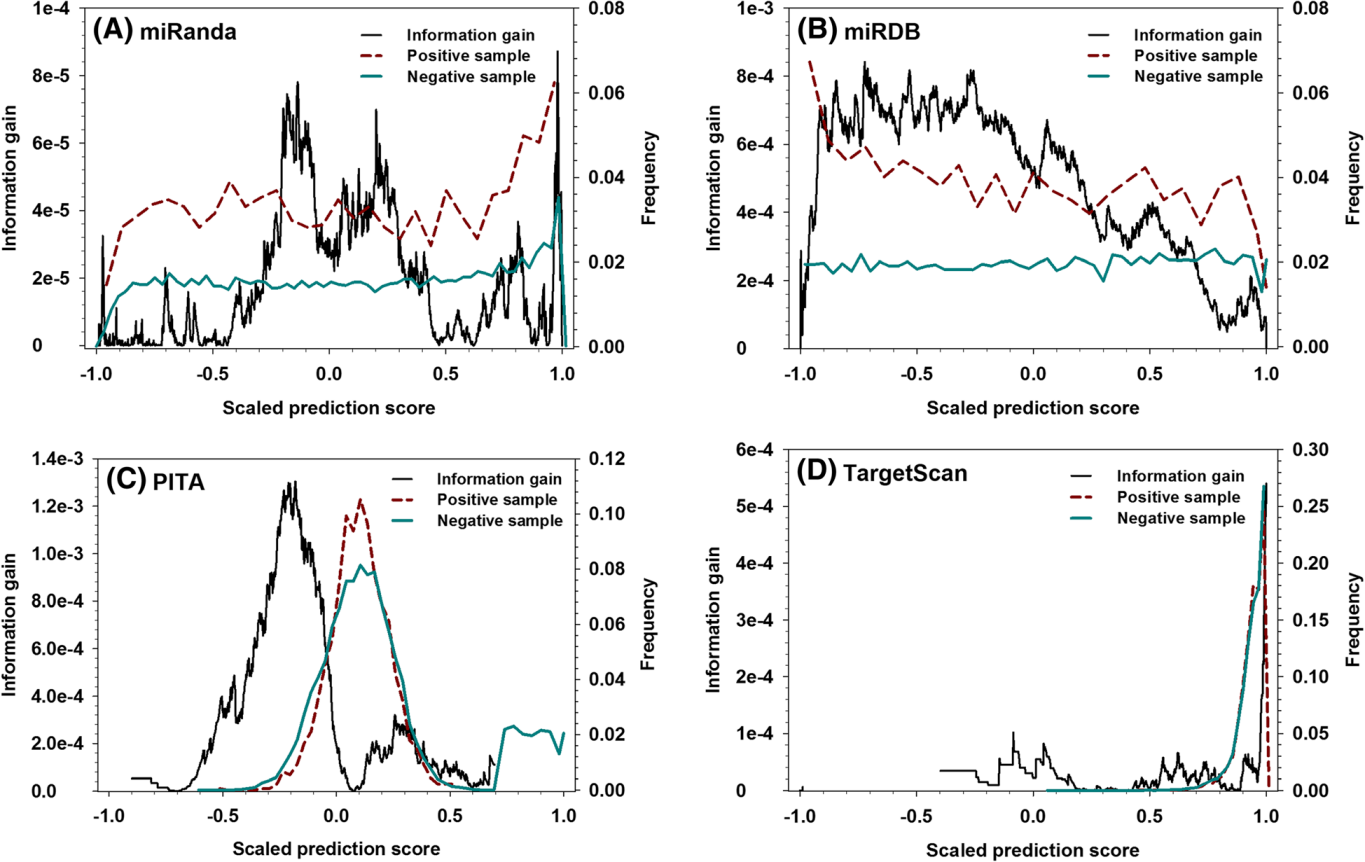
Information gain compared to the distribution of positive and negative samples in the D4 dataset for (**a**) miRanda, (**b**) miRDB, (**c**) PITA, and (**d**) TargetScan. X-axis shows the scaled prediction score, y-axis on the left shows the value of information gain, and y-axis on the right shows the distribution of positive samples (red dashed) and negative samples (cyan solid)

Figure 9 shows the information gain as a function of the scaled prediction score for each individual predictor in the D4 dataset, as well as the distribution of positive and negative samples at different prediction scores in the D4 dataset (see Additional file 1: Figure S2 for data in the other ten datasets). Clearly, the plots of information gain of four predictors are very different from the curves of distribution of positive and negative samples. In other words, information gain provides additional information that cannot be easily produced by the comparison of distribution between positive and negative samples. By notation, a spike in the plot of information gain indicates that the predictive score associated with this spike is able to split positive samples from negative samples in an optimal way. Therefore, all the spike-associated prediction scores are candidates of threshold values. By taking into consideration that positive samples’ prediction scores are smaller in the scaled prediction scores, the threshold values for true predictions should be smaller than the threshold values for false predictions. In addition, the 1st-step threshold values should be more stringent than the 2nd-step threshold values. With that said, the 1st-step threshold values for true predictions should be smaller than 2nd-step threshold values for true predictions, and the 1st-step threshold values for false predictions should be larger than the 2nd-step threshold values for false predictions. When selecting the parameters for each DANN-x module, an initial set of parameters that satisfy the afore-mentioned conditions were selected to evaluate the final prediction accuracy. At this point, normally the values associated with the largest four peaks were used, and the accuracies associated with the first-step true/false as well as the second-step true/false are calculated. Then, one of the threshold values was changed in a way satisfying the afore-mentioned criteria, and the prediction accuracy of this module is re-evaluated to compare with the previously calculated accuracy. If the parameter has been changed twice and the accuracy has been continuously decreasing, the selection of this parameter will be terminated and the associated threshold value will be determined. Apparently, the tuning of parameters has four subsequent phases: first-step true, second-step true, first-step false, and second-step false. If the change of first-step threshold value (or true/false threshold values) affected the corresponding second-level threshold value (or false/true threshold values), the second-level threshold value (or false/true threshold values) will also be changed accordingly. Consequently, multiple combinations of threshold values were tested, and the final determined true/false threshold values of miRanda, miRDB, PITA, and TargetScan in the D4 dataset are (− 0.179/0.844, − 0.702/0.059, − 0.449/0.257, 0.896/0.982) as the 1st-step threshold values, and (0.067/0.199, − 0.480/− 0.269, − 0.203/− 0.107, 0.964/0.972) as the 2nd-step threshold values (see Additional file 1: Table S1 for the final threshold values of the other ten datasets).

### Prediction performance evaluation

The performance of the newly designed meta-strategy was evaluated using Sensitivity (Sens), Specificity (Spec), Accuracy (Acc), F1 score (F1), and Matthews Correlation Coefficient (MCC) under multi-fold cross-validation and in independent datasets. The performance was further compared with the corresponding values of four individual predictors (miRanda, miRDB, PITA, and TargetScan), as well as another two recently developed meta-predictor: ComiR [30] and Oliveira’s predictor [55]. ComiR is a recently developed meta-predictor for miRNA target prediction. By using support vector machine to integrate the predictive results of miRanda [24], PITA [17], and TargetScan [12], ComiR improved the overall prediction accuracy remarkably, but still left room for further improvement.

## Additional file

Additional file 1: **Table S1.** The 1st-step and 2nd-step threshold values for both true and false predictions in eleven DANN modules. **Table S2.** Sensitivity (Sens), specificity (Spec), and accuracy (Acc) of miRanda, miRDB, PITA, TargetScan, MTR*, and ComiR in the eleven non-redundant datasets under multi-fold cross-validation. **Table S3.** F1 and Mathews Correlation Coefficient (MCC) of miRanda, miRDB, PITA, TargetScan, MTR*, and ComiR in the eleven non-redundant datasets under multi-fold cross-validation. **Table S4.** Sensitivity (Sens), specificity (Spec), and accuracy (Acc) of miRanda, miRDB, PITA, TargetScan, MTR*, and ComiR in the eleven independent test datasets. **Table S5.** F1 and Mathews Correlation Coefficient (MCC) of miRanda, miRDB, PITA, TargetScan, MTR*, and ComiR in the eleven independent test datasets. **Figure S1.** ROC curves of individual predictors in eleven newly designed datasets that contains duplicate samples. **Figure S2.** Information gain compared to the distribution of positive and negative samples in four D3 series datasets and six D2 series datasets for (A) miRanda, (B) miRDB, (C) PITA, and (D) TargetScan, when the prediction scores of these predictors are available. (DOCX 642 kb)

## Abbreviations

Acc: Accuracy
ANN: Artificial Neural Network
AUC: Area under the ROC curve
DANN: Decision-tree based Artificial Neural Network
F1: F1 score
HMM: Hidden Markov Model
MCC: Matthew’s Correlation Coefficient
ROC: Receiver operating characteristic
Sens: Sensitivity
Spec: Specificity
SVM: Support vector machine
