# Supplementary material for: Significant improvement of miRNA target prediction accuracy in large datasets using meta-strategy based on comprehensive voting and artificial neural networks
Source: BMC Genomics. 2019 Feb 27;20:158. doi: 10.1186/s12864-019-5528-1 (PMC6391818; doi:10.1186/s12864-019-5528-1)

**Supplementary tables and figures:**

**S. Table 1.** **The 1^st^-step and 2^nd^-step threshold values for both true and false predictions in eleven DANN modules.** Numbers above and below the slashes are for true and negative predictions, respectively. Values inside parentheses are negative values. “~~~” indicates the threshold values are not applicable in that module.


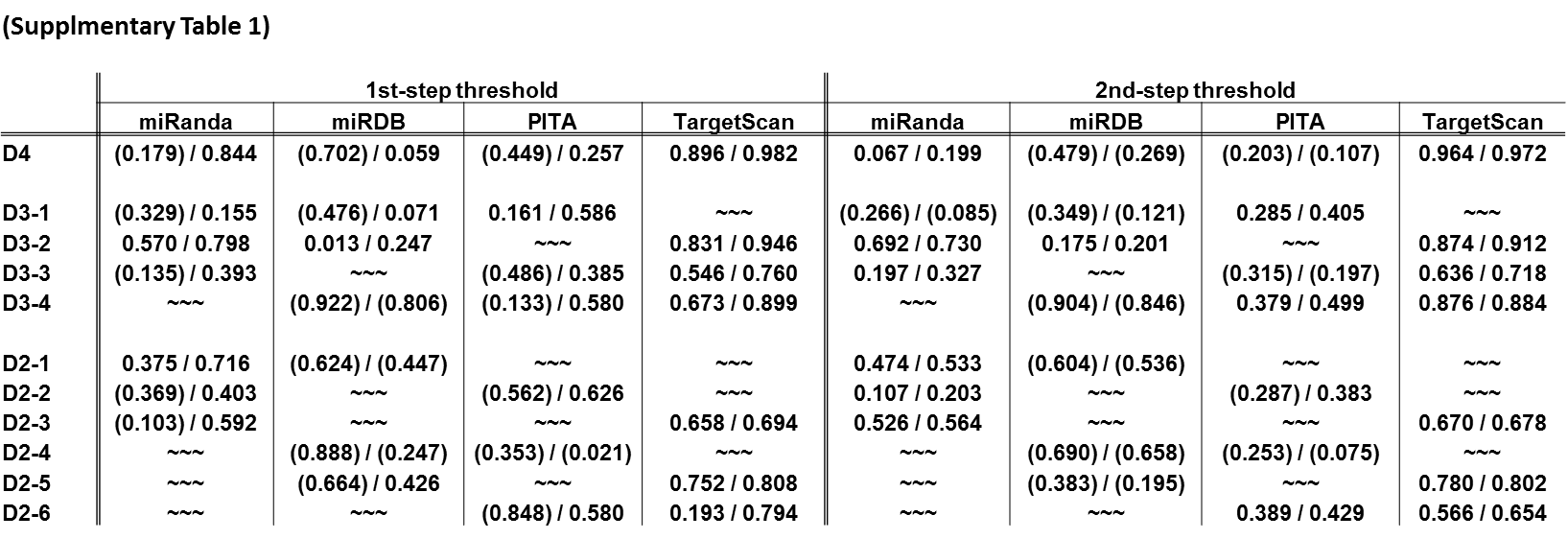


**S. Table 2. Sensitivity (Sens), specificity (Spec), and accuracy (Acc) of miRanda, miRDB, PITA, TargetScan, MTR*, and ComiR in the eleven non-redundant datasets under multi-fold cross-validation.** MTR* is a method developed by Oliveira, et. al. at 2017 (PMID:28559915). “~” indicates the value of a predictor in that dataset is not applicable. Values highlighted in red are the highest value in that category.

**S. Table 3.** **F1 and Mathews Correlation Coefficient (MCC) of miRanda, miRDB, PITA, TargetScan, MTR*, and ComiR in the eleven non-redundant datasets under multi-fold cross-validation.** MTR* is a method developed by Oliveira, et. al. at 2017 (PMID:28559915). “~” indicates the value of a predictor in that dataset is not applicable. Values in parentheses are negative values. Values highlighted in red are the highest value in that category.

**S. Table 4.** **Sensitivity (Sens), specificity (Spec), and accuracy (Acc) of miRanda, miRDB, PITA, TargetScan, MTR*, and ComiR in the eleven independent test datasets.** MTR* is a method developed by Oliveira, et. al. at 2017 (PMID:28559915). “~” indicates the value of a predictor in that dataset is not applicable. Values highlighted in red are the highest value in that category.

**S. Table 5.** **F1 and Mathews Correlation Coefficient (MCC) of miRanda, miRDB, PITA, TargetScan, MTR*, and ComiR in the eleven independent test datasets.** MTR* is a method developed by Oliveira, et. al. at 2017 (PMID:28559915). “~” indicates the value of a predictor in that dataset is not applicable. Values in parentheses are negative values. Values highlighted in red are the highest value in that category.

**S. Figure 1. ROC curves of individual predictors in eleven newly designed datasets that contains duplicate samples.** The datasets from top to bottom and from left to right are: D4, D3-1, D3-2, D3-3, D3-4, D2-1, D2-2, D2-3, D2-4, D2-5, and D2-6, respectively. Samples in the D4 dataset has prediction scores from four individual predictors, therefore, there are four ROC curves each for a predictor. Similarly, the D3 series datasets and D2 series datasets have three and two ROC curves, respectively. In each of the insets, x-axis shows the value of 1-specificity, while y-axis shows the values of sensitivity.


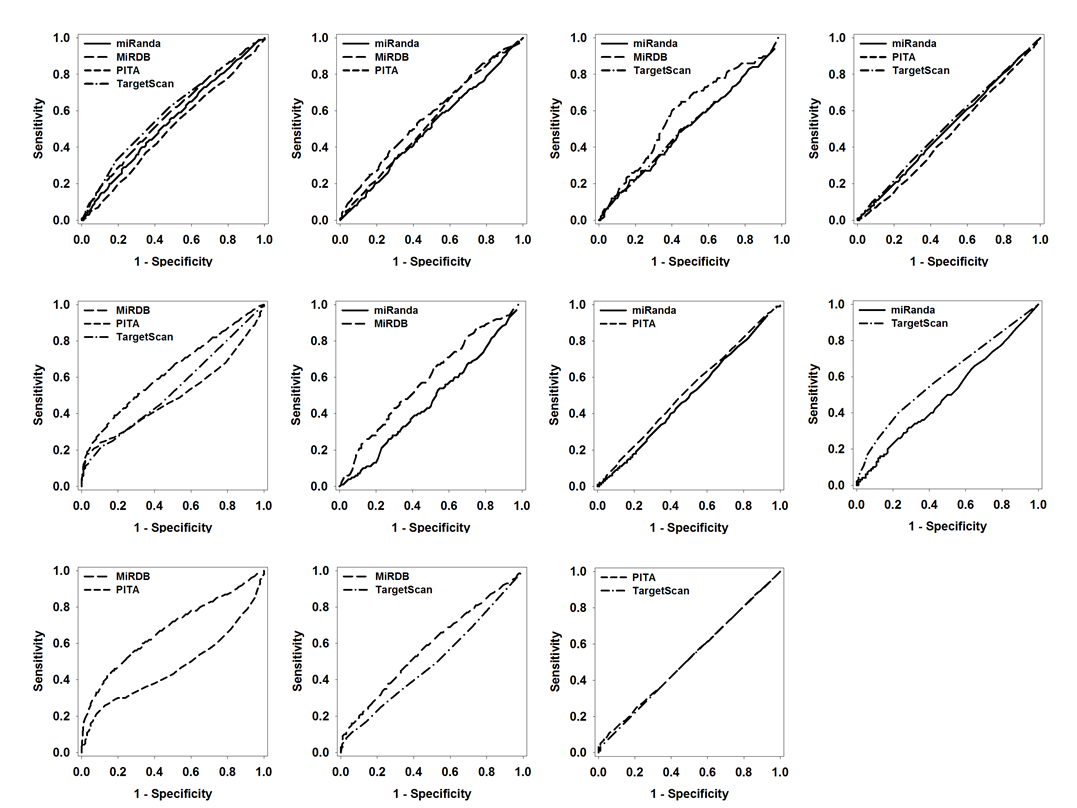


**S. Figure 2. Information gain compared to the distribution of positive and negative samples in four D3 series datasets and six D2 series datasets for (A) miRanda, (B) miRDB, (C) PITA, and (D) TargetScan, when the prediction scores of these predictors are available.** X-axis shows the scaled prediction score, y-axis on the left shows the value of information gain, and y-axis on the right shows the distribution of positive samples (red dashed) and negative samples (cyan solid).


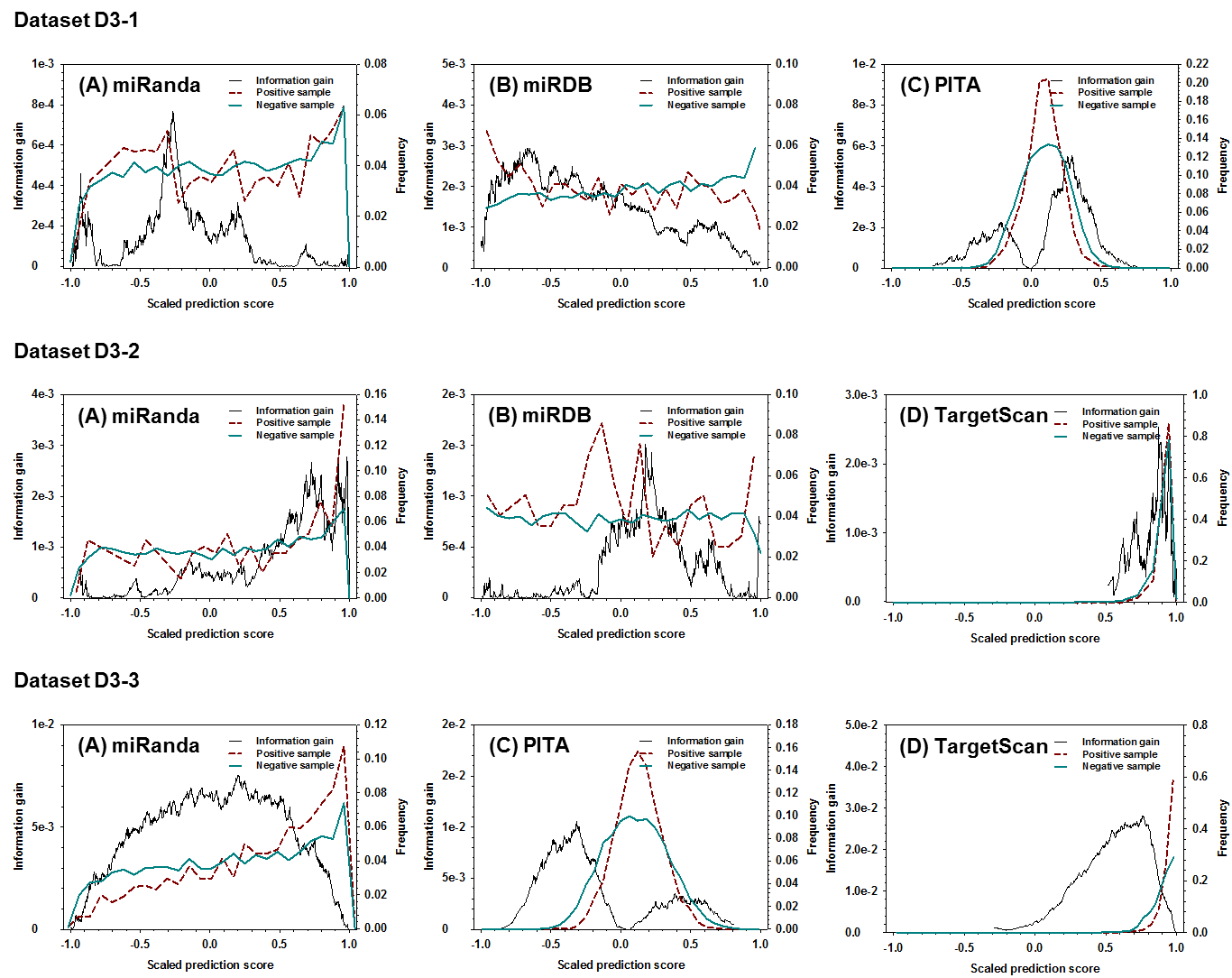


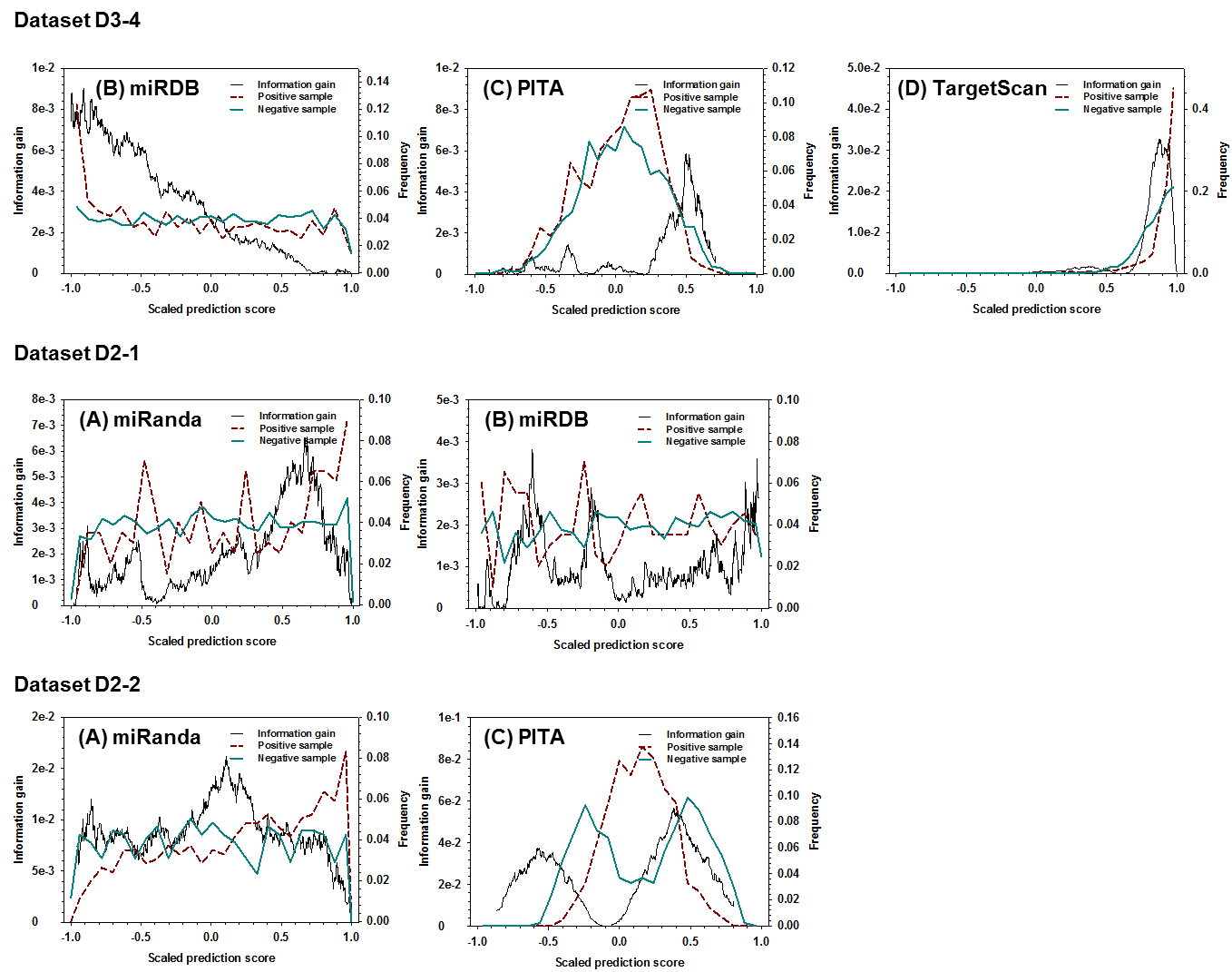


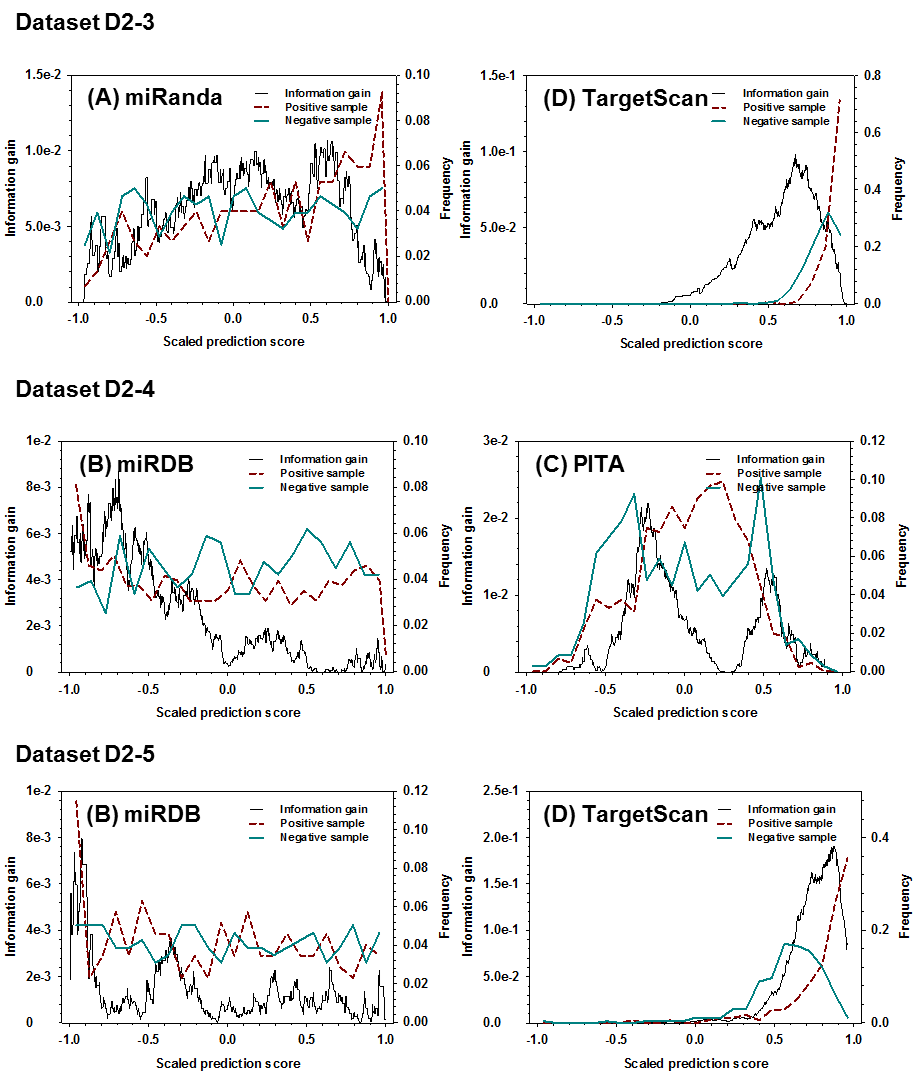


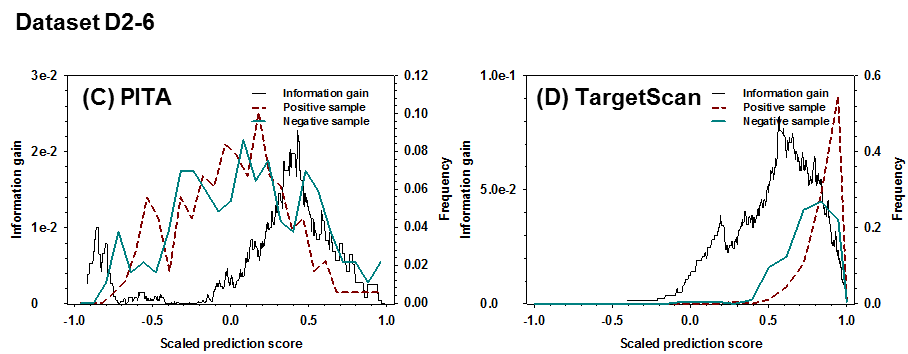

Supplement: Supplementary file 1 — Table S1. The 1st-step and 2nd-step threshold values for both true and false predictions in eleven DANN modules. Table S2. Sensitivity (Sens), specificity (Spec), and accuracy (Acc) of miRanda, miRDB, PITA, TargetScan, MTR*, and ComiR in the eleven non-redundant datasets under multi-fold cross-validation. Table S3. F1 and Mathews Correlation Coefficient (MCC) of miRanda, miRDB, PITA, TargetScan, MTR*, and ComiR in the eleven non-redundant datasets under multi-fold cross-validation. Table S4. Sensitivity (Sens), specificity (Spec), and accuracy (Acc) of miRanda, miRDB, PITA, TargetScan, MTR*, and ComiR in the eleven independent test datasets. Table S5. F1 and Mathews Correlation Coefficient (MCC) of miRanda, miRDB, PITA, TargetScan, MTR*, and ComiR in the eleven independent test datasets. Figure S1. ROC curves of individual predictors in eleven newly designed datasets that contains duplicate samples. Figure S2. Information gain compared to the distribution of positive and negative samples in four D3 series datasets and six D2 series datasets for (A) miRanda, (B) miRDB, (C) PITA, and (D) TargetScan, when the prediction scores of these predictors are available. (DOCX 642 kb) [file 12864_2019_5528_MOESM1_ESM.docx]
